# Supplementary material for: Glial insulin regulates cooperative or antagonistic Golden goal/Flamingo interactions during photoreceptor axon guidance
Source: eLife. 2021 Mar 5;10:e66718. doi: 10.7554/eLife.66718 (PMC7987344; doi:10.7554/eLife.66718)
Supplement: Supplementary file 1. [file elife-66718-supp1.docx]

**Supplementary Table 1 Related to Materials and Methods**

**List of genotypes used**

| Figure | Genotype (Genotypes unrelated to the context are in brackets.) | |
| --- | --- | --- |
| 1C | sensFLP / UAS-FLP; GMR-FsF-Gal4, UAS-myrRFP/+; gogo-FsF-GFP, sensGal4 / gogo-FsF-GFP | |
| 1D-1G | sensFLP ; gogo-FsF-GFP | |
| 1H | sensFLP ; fmi-FsF-mcherry ; sensGal4, UAS-mCD8GFP / + | |
| 1I-1L | sensFLP ; fmi-FsF-mcherry | |
| 1M, 1O | sensFLP ; fmi-FsF-mcherry ; gogo-FsF-GFP | |
| 2A, 2E, 2I | sensFLP ; GMR-FsF-Gal4/+; UAS-mCD8GFP / + | |
| 2B, 2F, 2J | sensFLP ; GMR-FsF-Gal4/UAS-gogoRNAi (GD3616) ; UAS-mCD8GFP / gogo[H1675] | |
| 2C, 2G, 2K | sensFLP ; GMR-FsF-Gal4/fmi[E59]; UAS-mCD8GFP / UAS-fmiRNAi (VDRC; GD607) | |
| 2O | sensFLP ; GMR-FsF-Gal4, UAS-FsF-GFP/+ ; tub-Gal80ts / + | |
| 2P | sensFLP ; GMR-FsF-Gal4, UAS-FsF-GFP/UAS-gogoRNAi (GD3616) ; tub-Gal80ts / gogo[H1675] | |
| 3A | Same as 2A, 2E, 2J | |
| 3B | sensFLP ; GMR-FsF-Gal4/UAS-gogoRNAi (GD3616) ; UAS-mCD8GFP / + | |
| 3C | sensFLP ; GMR-FsF-Gal4/+ ; UAS-mCD8GFP / UAS-fmiRNAi (VDRC; GD607) | |
| 3D | sensFLP ; GMR-FsF-Gal4/gogoRNAi (GD3616) ; UAS-mCD8GFP / UAS-fmiRNAi (VDRC; GD607) | |
| 3F | Same as 2A, 2E, 2J | |
| 3G | sensFLP ; GMR-FsF-Gal4/+; UAS-mCD8GFP / UAS-Gogo (V) | |
| 3I | sensFLP ; GMR-FsF-Gal4/+; UAS-mCD8GFP / UAS-Fmi | |
| 3J | sensFLP ; GMR-FsF-Gal4/UAS-gogoRNAi (GD3616); UAS-mCD8GFP / UAS-Fmi | |
| 3K | sensFLP ; GMR-FsF-Gal4/UAS-Gogo (V); UAS-mCD8GFP / UAS-Fmi | |
| 4A | Same as 1C | |
| 4B | sensFLP / UAS-FLP; GMR-FsF-Gal4, UAS-myrRFP/fmi[E59]; gogo-FsF-GFP, sensGal4 / gogo-FsF-GFP, UAS-fmiRNAi (VDRC; GD607) | |
| 4C | sensFLP / UAS-FLP; GMR-FsF-Gal4, UAS-myrRFP/+; gogo-FsF-GFP, sensGal4 / gogo-FsF-GFP, UAS-Fmi | |
| 4D | Same as 1C | |
| 4E | Same as 4C | |
| 4F | Same as 1H | |
| 4G | sensFLP ; fmi-FsF-ncherry / fmi-FsF-mcherry, UAS-gogoRNAi(GD3616) ; sensGal4, UAS-mCD8GFP / gogo[H1675] | |
| 4H | sensFLP ; fmi-FsF-mcherry ; sensGal4, UAS-mCD8GFP / UAS-Gogo (V) | |
| 4I | Same as 1C | |
| 4J | Same as 4C | |
| 5A | sensFLP ; GMR-Gal4/UAS-FsF-mCD8GFP; gogo[H1675] / gogo[D1600] | |
| 5B | sensFLP ; GMR-Gal4, UAS-GogoFL/UAS-FsF-mCD8GFP; gogo[H1675] / gogo[D1600] | |
| 5C | sensFLP ; GMR-Gal4, UAS-FsF-mCD8GFP/ UAS-GogoΔC; gogo[H1675] / gogo[D1600] | |
| 5D | sensFLP ; GMR-Gal4, UAS-GogoFFD/UAS-FsF-mCD8GFP; gogo[H1675] / gogo[D1600] | |
| 5E | sensFLP ; GMR-Gal4, UAS-GogoDDD/UAS-FsF-mCD8GFP; gogo[H1675] / gogo[D1600] | |
| 5G | sensFLP; GMR-Gal4 / + ; UAS-FsF-mCD8GFP / UAS-Fmi | |
| 5H | sensFLP; GMR-Gal4 / UAS-GogoFL; UAS-FsF-mCD8GFP / UAS-Fmi | |
| 5I | sensFLP; GMR-Gal4 / UAS-GogoΔC; UAS-FsF-mCD8GFP / UAS-Fmi | |
| 5J | sensFLP; GMR-Gal4 / UAS-GogoFFD; UAS-FsF-mCD8GFP / UAS-Fmi | |
| 5K | sensFLP; GMR-Gal4 / UAS-GogoDDD; UAS-FsF-mCD8GFP / UAS-Fmi | |
| 5M | Same as 5A | |
| 5N | Same as 5B | |
| 5O | Same as 5D | |
| 5P | Same as 5E | |
| 6A, 6B | sensFLP; GMR-FsF-Gal4/dinr[ex15]; UAS-mCD8GFP / dinrRNAi (BDSC 31037) | |
| 6D | y dilp6-Gal4/+; UAS-nlsGFP/+ | |
| 6E, 6F | yw; senslexA lexAopTomato/ato-t-myc ; loco-Gal4, UAS-mCD8GFP /UAS-shi[ts1] | |
| 6G | yw; senslexA lexAopTomato/ato-t-myc ; R85G01-Gal4, UAS-mCD8GFP /UAS-shi[ts1] | |
| 6H | yw; senslexA lexAopTomato/ato-t-myc ; R25A01-Gal4, UAS-mCD8GFP /UAS-shi[ts1] | |
| 6I | yw; senslexA lexAopTomato/Mz97-Gal4, UAS-stinger; ato-t-myc /UAS-shi[ts1] | |
| 6J | y dilp6-Gal4; senslexA lexAopTomato/+ ; + /UAS-shi[ts1] | |
| 6K | yw; senslexA lexAopTomato/dilp2-Gal4 ; + /UAS-shi[ts1] | |
| 6L | yw; senslexA lexAopTomato/hobRNAi (BDSC 66966); loco-Gal4, UAS-mCD8GFP /+ | |
| 6N | +/+; senslexA lexAopTomato | |
| 6O | +/+; senslexA lexAop-myrTomato/+; GMR-Fmi/+ | |
| 6P | y dilp6-Gal4; senslexA lexAop-myrTomato/+; GMR-Fmi/UAS-dilp6RNAi | |
| 7A, 7C | yw; senslexA lexAopTomato/+ ; loco-Gal4, UAS-mCD8GFP /+ | |
| 7B | UAS-FLP/yw; fmi-FsF-mcherry; loco-Gal4, UAS-mCD8GFP/+ | |
| 7D | yw; senslexA lexAopTomato/fmi[E59]; loco-Gal4, UAS-mCD8GFP / UAS-fmiRNAi (VDRC; GD607) | |
| 7F | sensFLP; fmi-FsF-mcherry/+; locoGal4, UAS-mCD8GFP/+ | |
| 7G | sensFLP; fmi-FsF-mcherry / fmi[E59] ; locoGal4, UAS-mCD8GFP/ UAS-fmiRNAi (VDRC; GD607) | |
| 7H | sensFLP; ; locoGal4/ gogo-FsF-GFP | |
| 7I | sensFLP; fmi[E59] / +; locoGal4/ gogo-FsF-GFP, UAS-fmiRNAi (VDRC; GD607) | |
|  |  | |
|  |  | |
| 1-S1 | sensFLP ; gogo-Gal4 / UAS-FsF-mCD8GFP | |
| 2-S1A | Rh6-GFP, eyFLP | |
| 2-S1B | Rh6-GFP, eyFLP; <gogo< /gogo[H1675] | |
| 2-S1C | Rh6-GFP, eyFLP; <fmi< / fmi[E59] | |
| 2-S1D | sensFLP ; GMR-Gal4/ + ; UAS-FsF-mCD8GFP / + | |
| 2-S1E | sensFLP ; GMR-Gal4/UAS-gogoRNAi(GD3616) ; UAS-FsF-mCD8GFP / gogo[H1675] | |
| 2-S1F | sensFLP ; GMR-Gal4/fmi[E59] ; UAS-FsF-mCD8GFP / UAS-fmiRNAi (VDRC; GD607) | |
| 2-S1G, I, K | Same as 2O | |
| 2-S1H, J, L | Same as 2P | |
| 3-S1A, E | Same as 2-S1D | |
| 3-S1B, F | Same as 2-S1E | |
| 3-S1C, K | Same as 2-S1F | |
| 3-S1D, H | sensFLP ; GMR-Gal4, fmi[E59]/ UAS-FsF-mCD8GFP, UAS-gogoRNAi(GD3616) ; UAS-fmiRNAi (VDRC; GD607) / gogo[H1675] | |
| 3-S1I | Same as 3I | |
| 3-S1J | Same as 3G | |
| 3-S1K | sensFLP ; GMR-FsF-Gal4, UAS-FsF-mCD8GFP / UAS-gogoRNAi(GD3616) ; UAS-Fmi / gogo[H1675] | |
| 3-S1L | sensFLP ; GMR-FsF-Gal4, UAS-FsF-mCD8GFP / fmi[E59] ; UAS-Gogo (V) / UAS-fmiRNAi (VDRC; GD607) | |
| 3-S1M | Same as 2A | |
| 3-S1N | Same as 3B | |
| 3-S1O | Same as 3I | |
| 3-S1P | Same as 3J | |
| 3-S2A | yw; senslexA lexAopTomato/+ ; gogo[H1675] /+ | |
| 3-S2B | yw; senslexA lexAopTomato/+ ; gogo[H1675] /gogo[D1600] | |
| 3-S2C | yw; senslexA lexAopTomato/+ ; gogo[H1675] /gogoΔGOGO1 | |
| 3-S2D | yw; senslexA lexAopTomato/+ ; gogo[H1675] /gogoΔGOGO2 | |
| 3-S2E | yw; senslexA lexAopTomato/+ ; gogo[H1675] /gogoΔGOGO3 | |
| 3-S2F | yw; senslexA lexAopTomato/+ ; gogo[H1675] /gogoΔGOGO4 | |
| 3-S2G | yw; senslexA lexAopTomato/+ ; gogo[H1675] /gogoΔCUB | |
| 3-S2H | yw; senslexA lexAopTomato/+ ; gogo[H1675] /gogoΔTSP1 | |
| 3-S2I | Same as 3I | |
| 3-S2J | sensFLP ; GMR-FsF-Gal4/GMR-GogoFL; UAS-mCD8GFP / UAS-Fmi | |
| 3-S2K | sensFLP ; GMR-FsF-Gal4/GMR-GogoΔN-D; UAS-mCD8GFP / UAS-Fmi | |
| 3-S2L | sensFLP ; GMR-FsF-Gal4/GMR-GogoΔN-E; UAS-mCD8GFP / UAS-Fmi | |
| 3-S2M | sensFLP ; GMR-FsF-Gal4/GMR-GogoΔN-G; UAS-mCD8GFP / UAS-Fmi | |
| 3-S2N | sensFLP ; GMR-FsF-Gal4/GMR-GogoΔN-H; UAS-mCD8GFP / UAS-Fmi | |
| 5-S1A | sensFLP ; GMR-FsF-Gal4/FRT42D hts null; UAS-mCD8GFP / htsRNAi (BDSC 35421) | |
| 5-S1C, D | sensFLP; GMR-Gal4 / + ; UAS-FsF-mCD8GFP / + | |
|  | sensFLP; GMR-Gal4, UAS-GogoFL / + ; UAS-FsF-mCD8GFP / + | |
|  | sensFLP; GMR-Gal4, UAS-GogoFFD / + ; UAS-FsF-mCD8GFP / + | |
|  | sensFLP; GMR-Gal4, UAS-GogoDDD / + ; UAS-FsF-mCD8GFP / + | |
|  | sensFLP; GMR-Gal4 / + ; UAS-FsF-mCD8GFP / UAS‐Add1‐myc | |
|  | sensFLP; GMR-Gal4 / UAS-GogoFL ; UAS-FsF-mCD8GFP / UAS‐Add1‐myc | |
|  | sensFLP; GMR-Gal4 / UAS-GogoFFD ; UAS-FsF-mCD8GFP / UAS‐Add1‐myc | |
|  | sensFLP; GMR-Gal4 / UAS-GogoDDD ; UAS-FsF-mCD8GFP / UAS‐Add1‐myc | |
| 5-S1F | GMR-gogoFFD T4a / + ; sens-Gal4, UAS-mCD8GFP / + |  |
|  | GMR-gogoDDD T1a / + ; sens-Gal4, UAS-mCD8GFP / + |  |
| 5-S1G | ey3.5FLP ; <fmiN< / fmi[E59], GMR-Gal4; UAS-FsF-mCD8GFP / + |  |
|  | ey3.5FLP ; <fmiN< / fmi[E59], GMR-Gal4; UAS-FsF-mCD8GFP / UAS-Fmi |  |
|  | ey3.5FLP ; <fmiN< / fmi[E59], GMR-Gal4; UAS-FsF-mCD8GFP / UAS-FmiΔC |  |
| 5-S1I | sensFLP ; GMR-FsF-Gal4 / + ; UAS-mCD8GFP / UAS-FmiΔC |  |
| 6-S1A | dilp1-Gal4 / + ; UAS-mCD8GFP / + |  |
| 6-S1B | dilp2-Gal4 / + ; UAS-mCD8GFP / + |  |
| 6-S1C | dilp3-Gal4 / + ; UAS-mCD8GFP / + |  |
| 6-S1D | dilp4-Gal4 / + ; UAS-mCD8GFP / + |  |
| 6-S1E | dilp5-Gal4 / + ; UAS-mCD8GFP / + |  |
| 6-S1F, H, I | y, dilp6-Gal4 / + ; UAS-mCD8GFP / + |  |
| 6-S1G | UAS-mCD8GFP / dilp7-Gal4 |  |
| 6-S1J | Act-Gal4, UAS-mCD8GF / + ; tub-Gal80ts/ dilp1RNAi |  |
|  | Act-Gal4, UAS-mCD8GF / + ; tub-Gal80ts/ dilp2RNAi |  |
|  | Act-Gal4, UAS-mCD8GF / + ; tub-Gal80ts/ dilp3RNAi |  |
|  | Act-Gal4, UAS-mCD8GF / + ; tub-Gal80ts/ dilp4RNAi |  |
|  | Act-Gal4, UAS-mCD8GF / + ; tub-Gal80ts/ dilp5RNAi |  |
|  | Act-Gal4, UAS-mCD8GF / + ; tub-Gal80ts/ dilp6RNAi |  |
|  | Act-Gal4, UAS-mCD8GF / + ; tub-Gal80ts/ dilp7RNAi |  |
|  | Act-Gal4, UAS-mCD8GF / dilp8RNAi ; tub-Gal80ts/ + |  |
| 7-S1A | 20C11FLP/GMR-FsF-Gal4 ; UAS-mCD8GFP / UAS-fmiRNAi (VDRC; GD607) |  |
| 7-S1B | senslexA lexAopTomato/fmi[E59] ; bshM-Gal4, UAS-myrGFP (from Kanazawa uni) / UAS-fmiRNAi (VDRC; GD607) |  |
| 7-S1C | 20C11FLP/GMR-FsF-Gal4 ; UAS-mCD8GFP / + |  |
| 7-S1D | 20C11FLP/GMR-FsF-Gal4, U-gogo (V) ; UAS-mCD8GFP / U-Fmi |  |
| 7-S1E | Rh4-GFP/+ |  |
| 7-S1F | Rh4-GFP/+ ; GMR-Gal4, U-Gogo(V)/+ |  |
| 7-S1G | Rh4-GFP/+ ; GMR-RhoA/+ |  |
| 7-S1H | Rh4-GFP/+ ; GMR-Gal4, U-Gogo(V)/+ ; GMR-RhoA/+ |  |
| 7-S1I | 20C11FLP/GMR-Gal4, U-gogo (V) ; UAS-FsF-mCD8GFP / U-Fmi |  |
| 7-S2B | w;UAS-mCD8GFP/+;; OK107-Gal4/+ |  |
| 7-S2C | w; UAS-mCD8GFP/+; gogo[H1675] / gogo[D1600]; OK107-Gal4/+ |  |
| 7-S2D | w; UAS-mCD8GFP/UAS-gogoFL; gogo[H1675] / gogo[D1600]; OK107-Gal4/+ |  |
| 7-S2F | w, UAS-dicer2/+;UAS-mCD8GFP/UAS-fmiRNAi;;OK107-Gal4/+ |  |
| 7-S2G | w, UAS-dicer2/+;UAS-mCD8GFP/+;UAS-Fmi/+;OK107-Gal4/+ |  |
| 7-S2H | UAS-dicer2/+;UAS-mCD8GFP/UAS-gogoRNAi;UAS-Fmi/+;OK107-Gal4/+ |  |
| 7-S2K | w;UAS-dicer/UAS-fmiRNAi;repo-Gal4/+; |  |
| control in 7-S2I | w, UAS-dicer2/+;UAS-mCD8GFP/40D-UAS;;OK107-Gal4/+ |  |
| gogo RNAi in 7-S2J | w, UAS-dicer2/+;UAS-mCD8GFP/UAS-gogoRNAi;;OK107-Gal4/+ |  |
|  |  |  |
| Movie gogo mutant | sensFLP ; GMR-Gal4 / UAS-FsF-mCD8GFP ; gogo-Flpstop / gogo[H1675] |  |
| Movie control | sensFLP ; GMR-Gal4 / UAS-FsF-mCD8GFP ; + / gogo[H1675] |  |
|  |  |  |
