# Supplementary material for: Glial insulin regulates cooperative or antagonistic Golden goal/Flamingo interactions during photoreceptor axon guidance
Source: eLife. 2021 Mar 5;10:e66718. doi: 10.7554/eLife.66718 (PMC7987344; doi:10.7554/eLife.66718)
Supplement: Supplementary file 2. [file elife-66718-supp2.docx]

**Supplementary Table 2**

**oligo DNAs used for generating and analyzing transgenic flies**

| CTTCGCAGAATATACCGCTCTTCC | Gogo gDNA1 GogoΔGOGO1 |
| --- | --- |
| AAACGGAAGAGCGGTATATTCTGC | Gogo gDNA1 GogoΔGOGO1 |
| CTTCGGAGCTAATTTACTGCGGCA | Gogo gDNA2 GogoΔGOGO1, ΔGOGO2 |
| AAACTGCCGCAGTAAATTAGCTCC | Gogo gDNA2 GogoΔGOGO1, ΔGOGO2 |
| CTTCGATTCCCGTGTGCGGATCAC | Gogo gDNA3 GogoΔGOGO2, ΔGOGO3 |
| AAACGTGATCCGCACACGGGAATC | Gogo gDNA3 GogoΔGOGO2, ΔGOGO3 |
| CTTCGCAGTACTCTACGTACTTCT | Gogo gDNA4 GogoΔGOGO3, ΔGOGO4 |
| AAACAGAAGTACGTAGAGTACTGC | Gogo gDNA4 GogoΔGOGO3, ΔGOGO4 |
| CTTCGCCACCGGTATCCGAGTCTG | Gogo gDNA5 GogoΔTSP1 |
| AAACCAGACTCGGATACCGGTGGC | Gogo gDNA5 GogoΔTSP1 |
| CTTCGTAAATGCAGTCCCACGTGT | Gogo gDNA6 GogoΔTSP1, ΔCUB |
| AAACACACGTGGGACTGCATTTAC | Gogo gDNA6 GogoΔTSP1, ΔCUB |
| CTTCGTTCTCTGCCGGCGATGGAC | Gogo gDNA7 GogoΔCUB |
| AAACGTCCATCGCCGGCAGAGAAC | Gogo gDNA7 GogoΔCUB |
| CTTCGCAGTACTCTACGTACTTCT | Gogo gDNA8 GogoΔGOGO4 |
| AAACAGAAGTACGTAGAGTACTGC | Gogo gDNA8 GogoΔGOGO4 |
| AAGCTGAAAAGTGCGAGAATGTCAAG | FW gogoFlpstop attB-UAS Tom |
| CATGGACTTGGACATCTGAGTGTTTG | REV gogoFlpstop attB-UAS Tom |
| TATAGCGGCCGCATTGAAGTTCCTATTCCGAAGTTCC | FW FRT |
| TATAACTAGTCAAAAGCGCTCTGAAGTTCCTATAC | REV FRT |
| TATAACTAGTTAAATCCAGACATGATAAGATACATTGATGAG | FW stop-FRT |
| TATACTGCAGCAAAAGCGCTCTGAAGTTCCTATAC | REV stop-FRT |
| GCGCCTCGAGAACTTCGTATAGCATACATTA | FW loxP-RFP-loxP |
| TATACTCGAGAACGTGTCGGTACCAATTGAGCTC | REV loxP-RFP-loxP |
| TATAGCATGCTGCACTGGACATCATTGAACTT | FW mini-white |
| TATAGAATTCCCAGTGAAATCCAAGCATTTTCTA | REV mini-white |
| TATAAAGCTTGGATCCGGCTTACCTTATCTGG | FW GogoFsFGFP pre |
| TATAGCTAGCCACGGCGACTTCCTTTGACTTC | REV GogoFsFGFP pre |
| TATACTCGAGCACCAAGATATAATTGTACATAAAACC | FW GogoFsFGFP post |
| TATAGGTACCTACAGGTCGGGGTGATATAGAAA | REV GogoFsFGFP post |
| TATACTGCAGATGAGTAAAGGAGAAGAACTTTTC | FW GFP |
| TATACTCGAGTCTAGTGGATCCAGACATGATAAG | REV GFP |
| CTTCGGAGCCGAAGTCAAAGGAAG | gogo gDNA Gogo-FsF-GFP |
| AAACTTCCTTTGACTTCGGCTCC | gogo gDNA Gogo-FsF-GFP |
| TATAAAGCTTAGCAGCACCACAACAAAATCAA | FW fmiFsFmcherry pre |
| TATAGCTAGCATATTCCGCCTCTGAGTCGGTAT | REV fmiFsFmcherry pre |
| TATACTCGAGAAAAGGTCTGCAGCAAGATTGTCC | FW fmiFsFmcherry post |
| TATAGGTACCCCATAGCATTTTGCATTACGTCGAA | REV fmiFsFmcherry post |
| TATAGTCGACATGGTGAGCAAGGGCG | FW mcherry |
| TATACTCGAGTCTAGTGGATCCAGACATGATAAG | REV mcherry |
| CTTCGACCTTTTGGCCAACTTACT | Fmi gDNA Fmi-FsF-mCherry |
| AAACAGTAAGTTGGCCAAAAGGTC | Fmi gDNA Fmi-FsF-mCherry |
